# Toxicity and bioconcentration of bisphenol A alternatives in the freshwater pulmonate snail *Planorbella pilsbryi*

**DOI:** 10.1007/s11356-025-36019-w

**Published:** 2025-02-05

**Authors:** Ève A. M. Gilroy, Karyn Robichaud, Maria Villella, Kara Chan, David W. G. McNabney, Carmen Venier, Victor Pham-Ho, Émilie C. Montreuil Strub, Shelby A. Ravary, Ryan S. Prosser, Stacey A. Robinson

**Affiliations:** 1 Aquatic Contaminants Research Division, Environment and Climate Change Canada, Burlington, ON Canada; 2 School of Environmental Sciences, University of Guelph, Guelph, ON Canada; 3 Ecotoxicology and Wildlife Health Division, Environment and Climate Change Canada, Ottawa, ON Canada

**Keywords:** Bisphenol A, Bisphenol F, Bisphenol S, Bisphenol AF, Freshwater snails, Aquatic toxicity

## Abstract

Bisphenol A (BPA) is an industrial chemical identified as a vertebrate endocrine disruptor. Numerous alternatives have been developed, for which toxicity data are lacking. The present study assessed the toxicity of BPA and its replacement products bisphenol F (BPF), bisphenol S (BPS), and bisphenol AF (BPAF) in freshwater snail (*Planorbella pilsbryi*) embryos and adults. The chronic toxicity of BPA and BPAF was further characterized in 28-day tests with adult snails, followed by 21-day assessments of hatching and survival of embryos produced at the end of the test (F1 generation). In acute tests, BPAF was the most toxic of the substances tested (maximum acceptable toxicant concentration [MATC], 136 µg/L), followed by BPA (MATC, 1404 µg/L), BPF (MATC, 1525 µg/L), and BPS (MATC > 8590 µg/L). In the chronic test with BPA, although we observed no significant effects on adult snails up to 479 µg/L, the hatching and survival of juveniles from the F1 generation decreased (MATC, 13 µg/L), and was delayed by 7.5 days, on average. In contrast, we did not observe any decrease in hatching or survival of juveniles from the F1 generation during exposure to BPAF. Effects were observed at concentrations above most reported environmental exposure concentrations, although there was an overlap between exposure and effect concentrations. Given that concentrations of alternative substances are expected to increase, and in the absence of data on potential effects of mixtures, further research is needed.

**Supplementary Information:**

The online version contains supplementary material available at 10.1007/s11356-025-36019-w.

## Introduction

Bisphenol A (BPA, 4,4′-isopropylidenediphenol) is an industrial chemical used as a precursor to plastic polymers from consumer products, including some food containers and/or packaging, adhesives and paper coatings, and others (Environment Canada and Health Canada 2008; Environment and Climate Change Canada 2017; Staples et al. 1998). In the last decades, BPA has been identified as an endocrine disrupting chemical in vertebrate models: the chemical structure of BPA enables it to bind to the estrogen receptors of vertebrates and mimic the effect of endogenous estrogens (Dodds and Lawson 1936; Environment and Climate Change Canada 2017). In 2008, the Government of Canada completed a screening assessment for BPA, which concluded that based on laboratory studies, low-concentration exposure to this contaminant could modify hormonal, developmental, or reproductive capacity in organisms in the environment, particularly at sensitive life stages. In addition, BPA was found to pose a risk to human health in Canada based on the potential for reproductive or developmental effects (Charkiewicz et al. 2024). Consequently, BPA has since been banned from polycarbonate baby bottles (Government of Canada 2010a), a decision followed by the European Union, and by voluntary market abandonment (Flint et al. 2012). BPA was added to the Toxic Substances List in Schedule 1 of the Canadian Environmental Protection Act 1999 (Government of Canada 2010b), yet despite these regulatory decisions targeting the highest risk for human health and environmental exposures in Canada, it remains in use in other plastic products that are not subject to BPA regulations, and numerous BPA alternatives have been developed. Several of these alternatives are similar in structure to BPA—for example, bisphenol F (BPF), bisphenol S (BPS), and bisphenol AF (Table 1)—and could pose similar health and environmental hazards (Moreman et al. 2017).

**Table 1:**
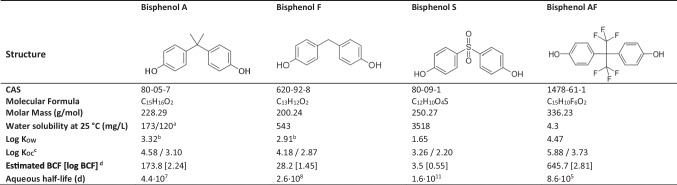
Structure and chemical properties of the bisphenol A, F, S, and AF, based on EPI Suite (US EPA 2012) unless stated otherwise

^a^Experimental data (Dorn 1987)

^b^Experimental data (Hansch et al. 1995)

^c^Molecular Connectivity Method/*K*_OW_ Method

^d^Estimates using the Arnos-Gobas Method

Studies have indicated that BPA and its replacement products leach from plastics and enter aquatic systems through wastewater treatment effluent and runoff from biosolid application to agricultural fields (Corrales et al. 2015; Flint et al. 2012). Environmental concentrations of BPA in surface waters are generally below 1 µg/L; in Canada, BPA has been measured most frequently at concentrations around 0.1 µg/L, with maximum effluent concentrations reaching 12 µg/L (Environment and Climate Change Canada 2017). More recently, in a study evaluating BPA concentrations in southern regions of Canada, concentrations as high as 6.37 µg/L were reported in surface water, 130 µg/kg dry weight in sediments, and 1940 µg/L in landfill leachate (Gewurtz et al. 2021). BPA alternatives BPF, BPS, and BPAF were also detected in surface water, sediment, and sewage effluent, at lower concentrations than BPA but within a similar order of magnitude (Fromme et al. 2002; Song et al. 2014; Yang et al. 2014). Considering the increasing use of BPA alternatives, concentrations of these compounds are expected to increase; moreover, little is known about the potential effects of co-occurring replacement substances, for which few data are available, particularly in non-mammalian species (Canesi and Fabbri 2015).

Freshwater invertebrates are often used in toxicological studies because they play an important role in ecosystems, represent an abundant food source to other organisms, and are involved in nutrient cycling (deFur 2004); they are also easy to culture in a laboratory and many have short life spans, which reduces the amount of time required for chronic or life-cycle assessments. Freshwater gastropods are generally sensitive to physical or chemical destruction of their habitat and are among the most imperiled taxa alongside freshwater mussels (Burch 1989; Johnson et al. 2013). The freshwater pulmonate snail *Planorbella pilsbryi* resides in the water column and the substrate of shallow waters of lakes and ponds of North America, feeding on algae and organic debris from various substrates (Burch 1989; Clarke 1981; Peckarsky et al. 1990). As is the case with most pulmonate freshwater snails, *P. pilsbryi* is a simultaneous hermaphrodite. Snails deposit clutches of egg masses onto solid substrates; reproduction in the wild is seasonal (e.g. from May to November), but when maintained under fixed laboratory conditions, seasonality disappears and the snails reproduce year-long. *P. pilsbryi* has been identified as a suitable test species for assessing environmental contamination in pulmonate gastropods (Prosser et al. 2017, 2016; Osborne et al. 2020; Osborne et al. 2023).

The objective of the present study was to determine the effects of BPA and selected common replacement substances BPF, BPS, and BPAF, on two life stages of the freshwater gastropod *Planorbella pilsbryi*. We assessed acute toxicity in 96-h static tests with adult snails, to determine effects on survival, behaviour, and reproductive output. We also assessed effects on the hatching of snail embryos (13-day static renewal tests). Lastly, we assessed the chronic toxicity of BPA and BPAF in 28-day tests with adult snails, and in addition to assessing the effects on survival, growth, and reproductive output, we prolonged the test for an additional 21 days to assess the hatching of the egg masses laid at the end of the test (days 24–28) and the survival post-hatch of F1 juveniles. Hence, we assessed several apical endpoints pertaining to successful reproduction (i.e. reproductive output, hatching, survival of F1 juveniles) to provide an environmentally meaningful indicator of population health with exposure to the selected bisphenol substances.

## Materials and methods

### Chemicals

Bisphenol A (4,4′-isopropylidenediphenol, CAS#80-05-7; ≥ 99% purity), bisphenol S (4,4′-sulfonyldiphenol, CAS#80-09-1; ≥ 98% purity), and bisphenol AF (4,4″-hexafluoroisopropylidenediphenol, CAS# 1478-61-1, 97% purity) were purchased from Sigma-Aldrich (Oakville, ON, Canada; now called Sigma Millipore). Bisphenol F (4,4′-Methylenediphenol, CAS#620-92-8; ≥ 96% purity) was purchased from Toronto Research Chemicals (Toronto, ON, Canada). The properties of bisphenol A and selected replacement substances are summarized in Table 1. For testing with adult snails, a stock of each solution was prepared in absolute ethanol (Greenfield Global, Mississauga, ON, Canada). Further testing with embryos was completed without solvents, as we observed bacterial growth during the adult tests, and feared it may be detrimental to testing in the smaller volumes (2 mL) of the culture plates used for embryo tests.

### Culturing methods

A continuous culture of File Ramshorn snails (*Planorbella pilsbryi*) was established in the Aquatic Life Research Facility, Canada Centre for Inland Waters (Burlington, ON, Canada). Snails were originally acquired from the Aquatic Toxicology Unit of the Ontario Ministry of the Environment, Conservation and Parks (Toronto, ON, Canada). The culture was maintained at the Aquatic Life Research Facility (ALRF) in approximately 75 L aquaria equipped with Aquaclear filtration units, and filled with dechlorinated water (aka ALRF water; pH 7.7 ± 0.38, hardness 126 ± 4.6 mg/L; alkalinity 91 ± 3.5 mg/L; calcium 36 ± 1.4 mg/L) further enriched in calcium carbonate (7 g CaCO_3_ per tank per week), in a temperature- and light-controlled room held at 22 °C with a photoperiod of 16 h light:8 h dark. The filtration units were cleaned weekly, and half of the water was replaced by suction to remove debris. Snails were fed 7 g of shrimp pellets three times a week and organic spinach leaves ad libitum.

### Toxicity tests

#### Acute tests with adult snails

Aqueous, 96-h static tests were conducted to assess the toxicity of BPA, BPF, BPS, and BPAF on the survival, behaviour, and reproductive output of freshwater snails. Range-finding tests were first completed (2–3 concentrations, 1 replicate per concentration) to select the concentration for the highest treatment of each compound. One test was then conducted per compound and included six replicates of a negative control (0.16 mg/L CaCO_3_-supplemented ALRF water), a solvent control (0.02% ethanol v/v), and a nominal concentration range between 10 and 3200 (BPAF), 5000 (BPA), or 10,000 µg/L (BPF, BPS). Each chemical stock was dissolved in CaCO_3_-supplemented ALRF water to yield the appropriate concentration with a concentration of ethanol of 0.02%. A positive control consisting of 10,000 µg/L BPA (in 0.02% ethanol) was also included in the BPF, BPS, and BPAF tests.

The tests were conducted in 1-L aerated glass jars filled with 950 mL CaCO_3_-supplemented ALRF water and capped with a plastic lid to minimize evaporation. The jars were held in a 22 °C temperature- and light-controlled room with a photoperiod of 16 h light:8 h dark. The test was designed based on the OECD (2016) *Lymnaea stagnalis* Reproduction Test. Individual adult snails (15.6 ± 1.87 mm) were gently wiped with a lint-free tissue to remove egg masses or juveniles and weighed, and shell diameter was measured at the widest distance from the lip to the top of the last whorl. Before the 96-h BPF, BPS, and BPAF tests were completed, we implemented a 72-h acclimation period to first assess snail fecundity. For the acclimation period, the snails were transferred one by one to each jar containing CaCO_3_-supplemented ALRF water 72 h before the start of the test, in a randomized complete block design, for a total of five snails per jar. After 72 h (day 0), the number of egg masses produced in each jar was counted, the jars were assigned to each treatment following a randomized complete block design according to reproductive output, to ensure a balanced distribution of fertility among treatment groups (OECD 2016), and the snails were transferred to their respective exposure jar. The snails were then added one by one to each jar. Each jar was fed 0.1 g of ground shrimp pellets and 1 organic spinach leaf at *T* = 0 and at 48 h. Tests were conducted under static conditions to reduce the volume of chemical waste generated. Water quality parameters (pH, dissolved oxygen, conductivity, and total ammonia) were measured at the beginning and end of each test (Table S1). Every day, each jar was inspected to count the number of egg masses produced and assess snail behaviour [active (attached to the jar surface and visible)/inactive (inside its shell and thus not attached to the jar surface), location in the jar]. At the end of each test, the adult snails were removed, assessed for survival, measured, weighed, removed from their shell, and flash-frozen in liquid nitrogen for future tissue chemical analysis. The number of egg masses produced was divided by the number of surviving snails and the observations of snail behaviour were converted to a percentage of active snails. Water samples were collected from each treatment at the beginning and end of each test and stored at 4 °C in the dark (BPA, BPF, BPS), or frozen at -20 °C (BPAF), pending chemical analysis. Testing was interrupted by the COVID-19 pandemic, during which laboratories were shut down for a prolonged period. Upon resumption of activities, chemical analysis within standard analytical timelines could not be guaranteed, hence the requirement to freeze samples from the BPAF exposure, which was completed in the fall of 2021, after a 2-year hiatus.

####  Chronic tests with adult snails

Chronic toxicity of BPA and BPAF was assessed in 28-day (adults) + 21-day (F1 embryos) aqueous tests with adult *P. pilsbryi* and their subsequent offspring, under static renewal conditions, for comparison with the adult 96-h test. The adult test was designed to follow the OECD (2016) *Lymnaea stagnalis* Reproduction Test, when possible. Five adult snails (15.3 ± 1.32 mm) were transferred one by one to each jar containing CaCO_3_-supplemented ALRF water and subjected to a 72-h acclimation period. The jars were assigned to each treatment following a randomized complete block design according to reproductive output, and the snails were transferred to their respective exposure jar. The tests included six replicates of a negative control (CaCO_3_-supplemented ALRF water), a solvent control (0.02% ethanol), and nominal concentrations between 0.01 and 100 µg/L (BPAF) or between 0.01 and 1000 µg/L (BPA). The BPAF test also included a positive control of 1000 µg/L BPA. Each jar was fed 0.1 g ground shrimp pellets and 1 organic spinach leaf twice weekly. A full water renewal was conducted twice weekly [approximately every 3.5 days (BPA test), or every 3–4 days (BPAF test)] by transferring the snails into a fresh exposure jar. Water quality parameters (pH, temperature, dissolved oxygen, conductivity, and total ammonia) were measured before and after water renewals (Table S1). Dead snails were removed from the jar at water renewal to preserve water quality. On day 28, surviving adult snails were counted, measured, and weighed. Their shells were removed, and their tissues were quickly frozen in liquid nitrogen for future tissue analysis. The number of egg masses produced was divided by the number of surviving snails and by the number of weeks to calculate reproductive output, and the observations of snail behaviour were converted to a percentage of active snails. Egg masses laid since the previous water renewal (days 24–28) were left in the jars, and the treatment water was renewed twice weekly for 21 days to assess hatching success. Each jar was fed 0.1 g of ground shrimp pellets after the first egg mass hatched. The number of hatching egg masses was recorded throughout, and the number of surviving juveniles was counted on day 21. Water samples were collected from each treatment before (composite residue sample) and after (stock) renewals and stored at 4 °C in the dark (BPA) or frozen at −20 °C (BPAF), pending chemical analysis, because the latter test took place during the COVID-19 pandemic, and standard timelines for analysis could not be guaranteed.

#### Tests with snail embryos

Testing with snail embryos was completed at the University of Guelph, using a *P. pilsbryi* culture of the same lineage as that from Burlington, with similar culturing conditions, and following the protocol designed by Osborne et al. (2023). For each test, adult snails (15.9 ± 1.72 mm) were transferred to glass containers lined with food-grade silicone sheets and kept overnight to allow them to lay egg masses. The next morning, the adults were removed, and egg masses were carefully peeled from the silicone sheets using a paintbrush, transferred to each well of a 24-well plate, and 2 mL of exposure solution was added. The test included a negative control (CaCO_3_-supplemented ALRF water) and five exposure treatments with nominal concentrations varying between 100 and 10,000 µg/L (BPA, BPF, BPS) or between 10 and 1000 µg/L (BPAF). Stock solutions corresponding to the highest concentration were prepared in ALRF water and mixed on a stir plate for 12 h. The treatment solutions were prepared by dilution prior to testing. No solute or precipitate was visible after mixing, suggesting full dissolution of the compounds. Each treatment contained four replicate egg masses. The test was run at 25 °C with a photoperiod of 16 h light:8 h dark. Solutions were made every 2 days, and exposure solutions were collected before use in the experiment (stock solutions) and after removal from the wells for solution renewal (composite sample at 48 h) and stored at −20 °C pending chemical analysis. Testing continued until >60% of the eggs had hatched in all replicates of the control or for 13 days, depending on which came first. Images of each egg mass were taken under a stereomicroscope (×10 magnification) until complete mortality occurred in a replicate or until the test was ended. Hatching was calculated as the total number of juveniles hatched divided by the total number of eggs.

### Chemical analysis

#### Water

Water samples for chemical analysis of BPA, BPF, BPS, and BPAF were brought to room temperature, diluted as needed, and spiked with 50 µL of internal standard. The analytes were separated using a Waters Xevo TQ-S UHPLC-MS/MS (before 2021) or a Sciex 6500+ QTRAP UHPLC (2021-present) system equipped with a Waters ACQUITY BEH C18 column (100 × 2.1 mm, 1.7 µm particle size) and the appropriate guard column, operated in negative Electrospray Ionization (ESI-). An in-line filter (Phenomenex KrudCatcher ULTRA HPLC in-line filter, 2.0 µm depth × 0.004 in ID) was used to ensure the protection of the analytical column from particulate matter. The mobile phase consisted of 70:30 acetonitrile:water at a flow rate of 400 µL/min and using an isocratic elution, to ensure that BPA contamination, inherent in many instrument components, be effectively integrated into the baseline of the chromatogram. Optimized ESI-MS/MS conditions, ion transitions, and internal standards used for each analyte are presented in Table S2 and Table S3.

#### Tissues

Frozen tissues were shipped on ice to SGS AXYS Analytical Services (Sydney, BC) via overnight courier, where they were extracted and analyzed using SGS AXYS method MLA-113. Briefly, samples were extracted by sonication with acetonitrile and phosphate buffer at pH = 2. The solvent was evaporated by rotary evaporation, and the aqueous extract was diluted to 200 mL with reagent water, pH adjusted to 1.5 to 2 and cleaned up by solid phase extraction using Waters Oasis HLB cartridges, then Waters MAX SPE cartridges. The samples were eluted with methanol, concentrated, and filtered through a 0.2-μm PTFE filter. For samples analyzed prior to 2023, instrumental analysis was performed using a Waters 2795 HPLC connected via an electrospray interface to a Waters Quattro Ultima MS/MS operated in negative ESI running in multiple MRM mode. For samples analyzed in 2023 (tissue samples from the BPAF chronic test), instrumentation was upgraded to Waters TQ-S Xevo UPLC MS/MS, equipped with Acquity UPLC I-class sample manager. Analyte separation was achieved on a Waters Xterra C18 MS column (100 mm × 2.1 mm, 3.5 μm) maintained at 40 °C. The mobile phase consisted of methanol (solvent A) and HPLC water adjusted to pH = 10 (solvent B) set at a constant flow rate of 0.2 L/min. Quantification was achieved by isotope dilution/recovery correction quantification. An 8-point calibration range, with calibration verification at calibration point 6, was applied using linear regression equations with 1/*x* weighting and excluding the origin.

### Statistical analysis

Differences among treatments were tested using a single factor analysis of variance (ANOVA). When the ANOVAs revealed significant differences (*α* < 0.05), Dunnett’s post hoc test was performed to identify treatments that differed from controls. When applicable, if no differences between the control and solvent control were detected, the two controls were pooled; if differences existed, comparisons were made to the solvent control (Green 2014). In case of violation of the assumptions of normal distribution of residuals and homoscedasticity, differences were tested using the Kruskal-Wallis test, followed by Dunn’s multiple comparisons to determine differences from controls. Analyses were completed using Microsoft Excel and Sigma Plot (ver. 13.0). EC_50_ values for hatching success were calculated using the best-fitting model among 3- or 4-parameter log-logistic or Weibul models (constrained between 0 and 100, as needed) using the statistical package *drc* v.1.1.456 (Ritz et al. 2015) in R v .4.1.0 (R Core Team 2021). When the data were not amenable to calculating ECx values, the lowest-observed effect concentrations (LOECs; the lowest concentration at which significant effects were observed) and no-observed effect concentrations (NOECs; the lowest concentration at which no significant effects were observed) were used to calculate maximum acceptable toxicant concentrations (MATCs; the geometric means of the LOEC and NOEC), using the equation MATC=√(LOEC)(NOEC). For the calculation of bioconcentration factors (BCF), the mean measured tissue concentrations and mean water concentration for the duration of the exposure were used in the equation BCF = [tissue ng/g ww] / [mean water µg/L] for final BCF values in litres per kilogram. All data are reported based on measured values. 

## Results

### Chemical analysis

#### Water samples

The results of the chemical analyses are summarized in Table 2 and Table 3. In the 96-h tests with adult snails, spiked concentrations were 80–100% of nominal concentrations. We observed considerable breakdown of BPA and BPF: concentrations of BPA decreased to 30–60% within 96 h; BPF concentrations decreased to 12% or less. The concentrations of BPS remained within 74–89% of nominal concentrations after 96 h (Table 2). The lowest BPA concentration from the 96-h exposure, 0.01 µg/L (nominal), was below detection limit in the stock solution, and was measured as 0.072 µg/L in the residue. In the absence of two measurements to calculate a mean, we report the value as 0.072 µg/L.

**Table 2:** Measured concentrations of bisphenol A, F, S, and AF in water and tissue samples during aqueous 96-h static tests with the freshwater snail *Planorbella pilsbryi*. When more than one measurement is presented, the number of samples is included in parentheses. The mean ± standard deviation is reported when three values are available. *n.m*., not measured; *LOD*, limit of detection

Chemical tested	Treatment/nominal concentration (µg/L)	Water (µg/L)		Tissue (ng/g ww)	BCF (L/kg)
		Day 0	96 h	96 h	
Bisphenol A	Control	<LOD	<LOD	< LOD (3)	
	Solvent control	<LOD	0.11	< LOD (3)	
	0.01	<LOD	0.07	< LOD (3)	
	0.1	0.0684	0.10	n.m.	
	1	0.995	0.30	<LOD—7.62 (3)^a^	
	10	4	3	n.m.	
	100	97	36	551 ± 350.0 (3)	8.3
	1000	968	417	n.m.	
	5000	5390	2950	n.m.	
Bisphenol F	Control	0.08	<LOD^c^	n.m.	
	Solvent control	0.16	0.10	< LOD (3)	
	0.01	0.16	<LOD	n.m.	
	0.1	0.24	0.10	n.m.	
	1	0.82	<LOD	< LOD (3)	
	10	9^b^	0.06^c^	< LOD (3)^d^	
	100	92	0.12^e^	117 ± 158.1 (3)	2.5
	1000	933	19	n.m.	
	10,000	8550	1220	n.m.	
	BPA (10,000)	10,200	8370	n.m.	
Bisphenol S	Control	0.96	0.03	n.m.	
	Solvent control	0.04	0.03	< LOD-2.54 (3)^a^	
	0.01	0.211	0.04	n.m.	
	0.1	0.633^f^	0.10	n.m.	
	1	1	0.74	5 ± 2.4 (3)	5.4
	10	9	8	25 ± 4.3 (3)	3.0
	100	96	83	277 ± 27.6 (3)	3.1
	1000	854	833	n.m.	
	10,000	8270	8910	n.m.	
	BPA (10,000)	8580	5490	n.m.	
Bisphenol AF	Control	34	0.05	n.m.	
	Solvent control	0.05	0.06	12 ± 7.8^g^	n.m.
	0.01	0.5	0.43	n.m.	
	0.1	0.378	0.09	n.m.	
	1	2	2	25 ± 8.4 (3)^g^	12.7
	10	8	2	137 ± 9.7 (3)^g^	26.1
	100	69	6	1614 ± 1202.9 (3)	43.1
	1000	724	248	n.m.	
	3200	2460	1400	n.m.	
	BPA (10,000)	10,300	4080	n.m.	

Method detection limits (aqueous)—BPA, 0.0174 µg/L; BPF, 0.0159 µg/L; BPS, 0.0059 µg/L; BPAF, 0.0028 µg/L

Method reporting limit (tissue): BPA, 4–11.1 ng/g ww; BPF, 4.77–7.87 ng/g ww; BPS, 0.639–0.868 ng/g ww; BPAF, 2.16–2.77 ng/g ww—reporting limits depend on sample size

^a^Two samples were below detection limit

^b^0.499 µg/L BPA was also detected in the sample

^c^0.0442 µg/L BPS was also detected in the sample

^d^107 ± 89.5 (3) ng/g BPA was also detected in tissue samples

^e^0.0432 µg/L BPS was also detected in the sample

^f^0.020 µg/L BPF and 0.006 µg/L BPAF were also detected in the sample

^g^BPAF (15.8 ng/g ww) was detected in the associated blank, and the concentration in the sample was less than 10× the concentration detected in the blank

**Table 3:** Measured water concentrations of bisphenol A and AF during chronic aqueous 28-day + 21-day static renewal tests with adult freshwater snails (*Planorbella pilsbryi*) and the juveniles (F1) produced. When more than one measurement is presented, the number of samples is included in parenthesis. The mean ± standard deviation is reported when three values are available

Test	Treatment	Water (µg/L)		Tissue (ng/g ww)	BCF (L/kg)
		Stock	Residue (≈3.5–4 days)	28 days	
Bisphenol A	Control	<LOD (2)	<LOD (2)	< LOD (3)	
	Solvent control	<LOD (2)	<LOD–0.0225 (2)	< LOD (3)	
	0.01	<LOD (2)	<LOD–0.0188 (2)	< LOD (3)	
	0.1	<LOD–0.211 (2)	<LOD–0.0589 (2)	n.m.	
	1	0.773–0.748 (2)	0.0341–0.24 (2)	≤ LOD–10.3 (3)^a^	
	10	5.77–6.28 (2)	0.354–2.75 (2)	n.m.	
	100	64.6–70.7 (2)	8.52–23.5 (2)	581 ± 237.5 (3)	13.9
	1000	579–679 (2)	145–512 (2)	n.m.	
Bisphenol AF	Control	0.0274	0.0158	< LOD (2)	
	Solvent control	0.0189	0.0105	< LOD (3)	
	0.01	0.0326	0.0211	n.m.	
	0.1	0.127	0.0116	n.m.	
	1	1.07	0.0442	18 ± 10.8 (3)	32.3
	10	9.44	0.533	161 ± 57.4 (3)	32.3
	100	86.8	11.1	3170 ± 1015.0 (3)	64.8
	BPA (1000)	928	166	n.m.	

Method detection limit (aqueous): BPA, 0.0174 µg/L; BPAF, 0.0028 µg/L

Method reporting limit (tissue): BPA, 2.27–10 ng/g ww; BPAF, 6.3–14 ng/g ww

^a^One sample was below the method detection limit of 10 ng/g ww

In the 28-day test, concentrations of BPA at day 0 appeared lower than nominal concentrations (about 58–78%), which may indicate some breakdown in the refrigerated ethanol stocks. Concentrations of BPAF at day 0 were within the expected nominal concentrations (93–107%); there seemed to be some contamination (10–27 ng/L BPAF) in the control and solvent control. In the embryo tests, concentrations of BPA, BPF, and BPAF were also lower than expected; in the absence of solvent, all three compounds were tested at maximum concentrations that were above their predicted water solubility. In contrast, BPS was tested below its predicted solubility, and concentrations were similar to nominal concentrations, and did not break down over the 48 h between renewals (Table 4).

**Table 4:** Measured concentrations of bisphenol A, F, S, and AF in water during aqueous 13-day static-renewal tests with snail embryos (*Planorbella pilsbryi*)

Chemical tested	Treatment	Water (µg/L)	
		Day 0	48 h
Bisphenol A	Control	0.015	2
	100	<LOD	<LOD
	320	258	<LOD
	1000	513	4
	3200	int^a^	1180
	10,000	5530	50
Bisphenol F	Control	0.055	<LOD
	100	68	0.006
	320	127	0.14
	1000	559	4
	3200	2050	80
	10,000	6250	194
Bisphenol S	Control	0.004	1.91
	100	99	113
	320	337	347
	1000	970	939
	3200	2950	3180
	10,000	9890	12,000
Bisphenol AF	Control	<LOD	0.030
	10	9	5
	32	23	11
	100	70	39
	320	220	150
	1000	432	481

Method detection limits—BPA, 0.0174 µg/L; BPF, 0.0159 µg/L; BPS, 0.0059 µg/L; BPAF, 0.0028 µg/L

^a^Interferences prevented sample quantitation

#### Tissue samples

Of the tissues submitted for analysis, BPA was detected in snails exposed to nominal concentrations of 100 µg/L BPA, but not to 1 or 0.01 µg/L BPA (nominal; Table 2, Table 3). BCFs for BPA were 8.3 and 13.9 L/kg for the 96-h and 28-day tests, respectively. The BCF for BPF was 2.5 L/kg, the BCF for BPS varied between 3.0 and 5.4 L/kg, and the BCF of BPAF varied between 12.7 and 43.1 L/kg for the 96-h test (Table 2), and between 32.2 and 64.2 L/kg for the 28-day test (Table 3).

### Toxicity tests

#### Acute tests with adult snails

**Fig. 1:**
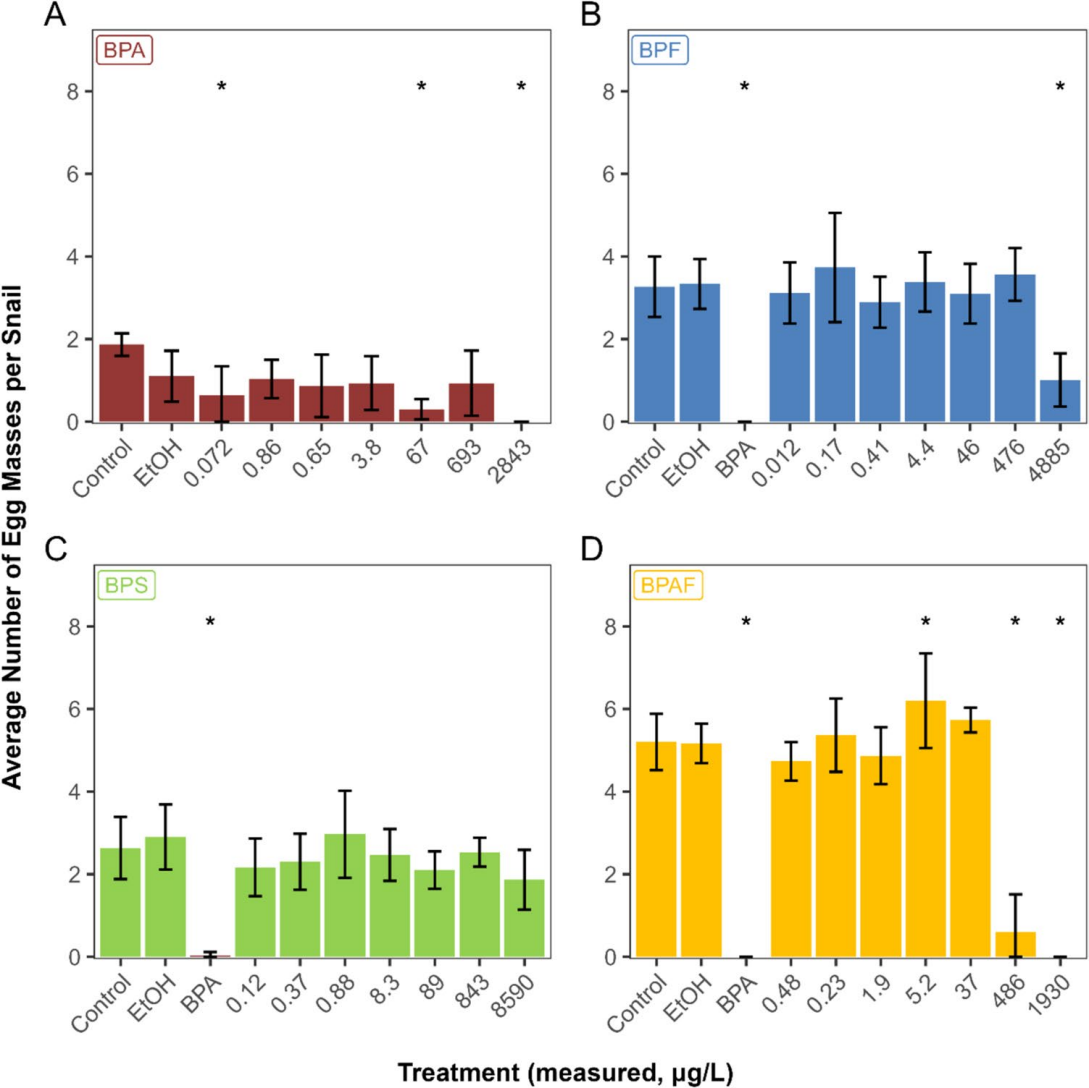
Effect of bisphenols on the reproductive output of adult freshwater snails *Planorbella pilsbryi* after a 96-h exposure (mean ± standard deviation, *n* = 6). **A** Bisphenol A. **B** Bisphenol F. **C** Bisphenol S. **D** Bisphenol AF. EtOH, ethanol (solvent) control; BPA, bisphenol A (positive) control at 10,000 µg/L (nominal). The reported concentrations correspond to mean measured concentrations in the solutions at the beginning of the experiment, and at the end of the 96-h exposure. Asterisks denote a significant difference from pooled controls

The 96-h exposure to BPA caused significant decreases in the survival of adult *P. pilsbryi* at 2842 µg/L, where only 30% of the snails survived, and 2740 µg/L BPAF, where no snails survived (Table S4). Survival was ≥ 80% in all other treatments. Exposure to BPF and BPS did not cause any mortality at concentrations up to 9285 µg/L (BPF) and 7035 µg/L (BPS), but significant mortality was observed in the BPA positive controls (0–33% survival; data not shown). We observed decreased activity in the snails exposed to the highest treatments of BPF (70% activity) and BPAF (58% activity), but not in those exposed to BPS (Table S4). Snail reproductive output was the most sensitive endpoint, and was affected by exposure to BPA, BPF, and BPAF, but not BPS: snails exposed to the highest BPA treatment did not produce any egg masses, those exposed to the highest BPF treatment produced 1 egg mass/snail over the 96-h period, and those exposed to the highest BPAF treatment, 0.6 egg mass/snail (Fig. 1). Reproductive output in the positive control was also significantly decreased (0–0.03 egg masses/snail; Fig. 1). The reproductive output from the snails exposed to BPA was variable and decreased significantly at exposure concentrations of 0.072 µg/L (measured below LOD in the stock solution but 0.072 µg/L in residue) and 67 µg/L (Fig. 1). Conversely, we observed a slight increase in the number of egg masses produced per snail in the 5.2 µg/L BPAF treatment. The NOECs, LOECs, and MATCs are summarized in Table 5.

**Table 5:** Toxicity of bisphenol A, F, S, and AF to *Planorbella Pilsbryi* in aqueous 96-h static tests (adults) and 28-day static renewal tests (adults (F0)) followed by a 21-day incubation and hatching period (juveniles (F1)), expressed as lowest-observed effect concentration (LOEC), no-observed effect concentration (NOEC), and maximum acceptable toxicant concentration (MATC). Concentrations are reported as micrograms per litre (measured)

Life stage	Test duration	Compound	Lowest-observed effect concentration	No-observed effect concentration	Maximum acceptable toxicant concentration (MATC)^a^	Most sensitive endpoint
Adults	96 h	Bisphenol A	2843	693^b^	1404	Reproductive output
		Bisphenol F	4885	476	1525	
		Bisphenol S	> 8590	8590	> 8590	
		Bisphenol AF	486	38	136	
Adults	28 + 21^c^ days	Bisphenol A	42	4	13	Percent hatching and number of juveniles
		Bisphenol AF	> 49	49	> 49	N/A^c^

^a^The maximum acceptable toxicant concentration (MATC) was calculated as the geometric mean of the LOEC and NOEC

^b^A significant decrease in reproductive output was noted at 42 µg/L BPA, which, in the absence of a concentration response, would require further research to assign causality

^c^There were no significant differences between treatments during the BPAF exposure

#### Chronic tests with adult snails

**Fig. 2:**
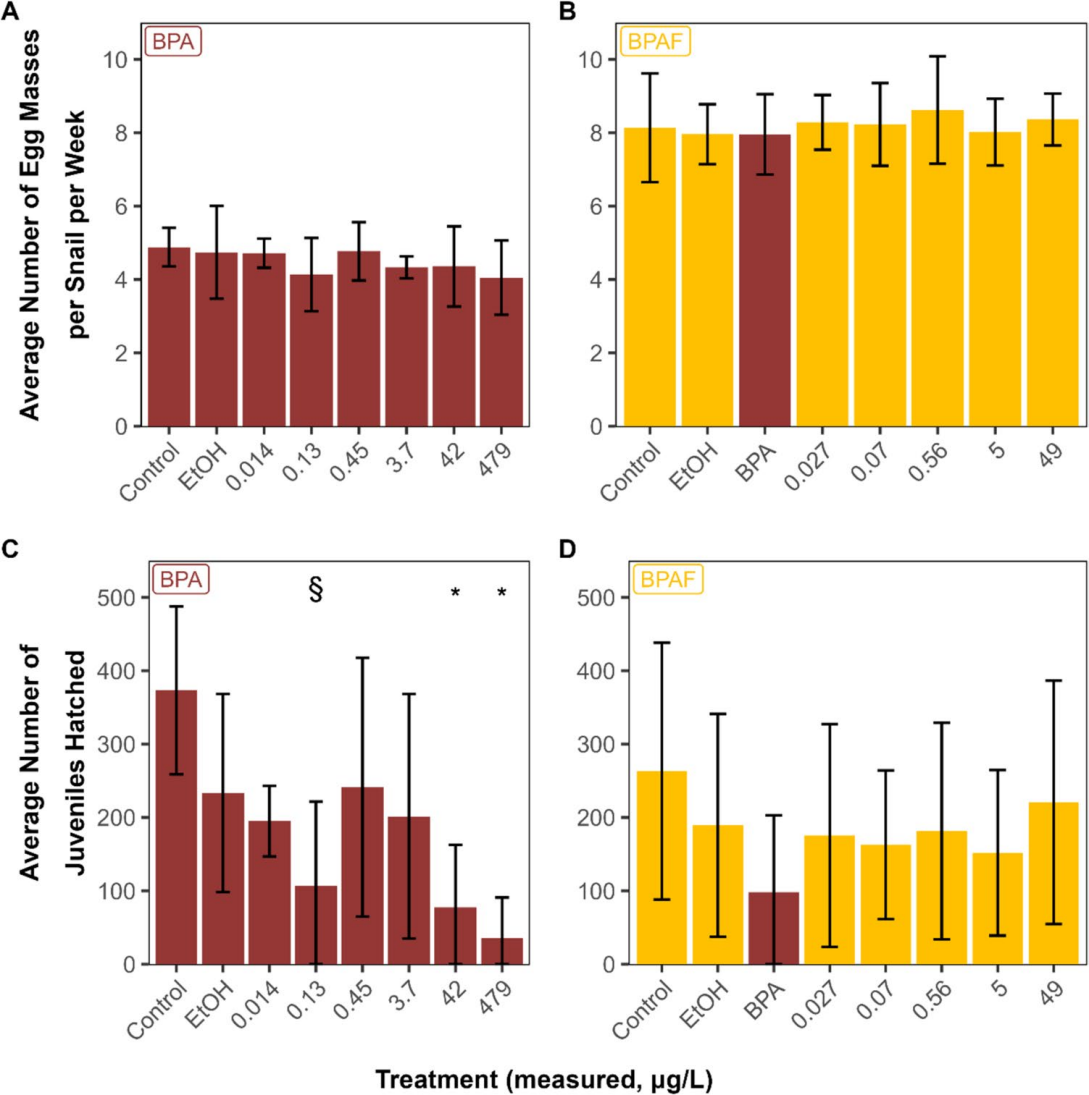
Reproductive output (**A**, **B**) and number of hatched juveniles (**C**, **D**) produced on days 25–28 (mean ± standard deviation, *n* = 6) after a 28-day exposure of adult freshwater snails *Planorbella pilsbryi* to bisphenol A (**A**, **C**) or bisphenol AF (**B**, **D**), based on mean concentrations measured in stock solutions and residues prior to water renewal. The reported concentrations correspond to mean measured concentrations in the stock solutions and in residues prior to water renewal. EtOH, ethanol (solvent) control. An asterisk denotes a significant difference from pooled controls; a section sign denotes a significant difference from the control, but not the solvent control or pooled controls

In 28-day exposures to BPA and BPAF, exposure to up to 479 µg/L BPA and 547 µg/L BPAF had no significant effects on survival, behaviour, or reproductive output (Table S5, Fig. 2). There were no differences in length or weight on either day 3 (*p =* 0.99, data not shown) or day 28 (*p* ≥ 0.13; Table S5), but the snails exposed to 0.13 µg/L BPA had slower growth than control snails (3.2 ± 1.44% (*n* = 6) vs. 6.7 ± 2.33% (*n* = 12, pooled controls; *p* = 0.01)). There was no relationship between snail size and number of juveniles produced (data not shown). In addition, although the number of egg masses produced did not differ from controls, we detected significant differences in the hatching success and number of F1 juveniles produced from the egg masses from the 42 and 479 µg/L BPA treatments laid at the end of the experiment (days 24–28; Fig. 2). The number of F1 juveniles from the 0.13 µg/L BPA treatment was also smaller than the control, but not the solvent control or the pooled controls (Fig. 2). Juveniles exposed to 479 µg/L BPA also hatched, on average, 7.5 days later than the pooled controls. In contrast, we did not observe any differences in the reproductive output, hatching success, or in the number of F1 juveniles from egg masses laid during the BPAF exposure (Fig. 2, Table S5). The NOECs, LOECs, and MATCs for all exposures are summarized in Table 5.

#### Tests with snail embryos

**Fig. 3:**
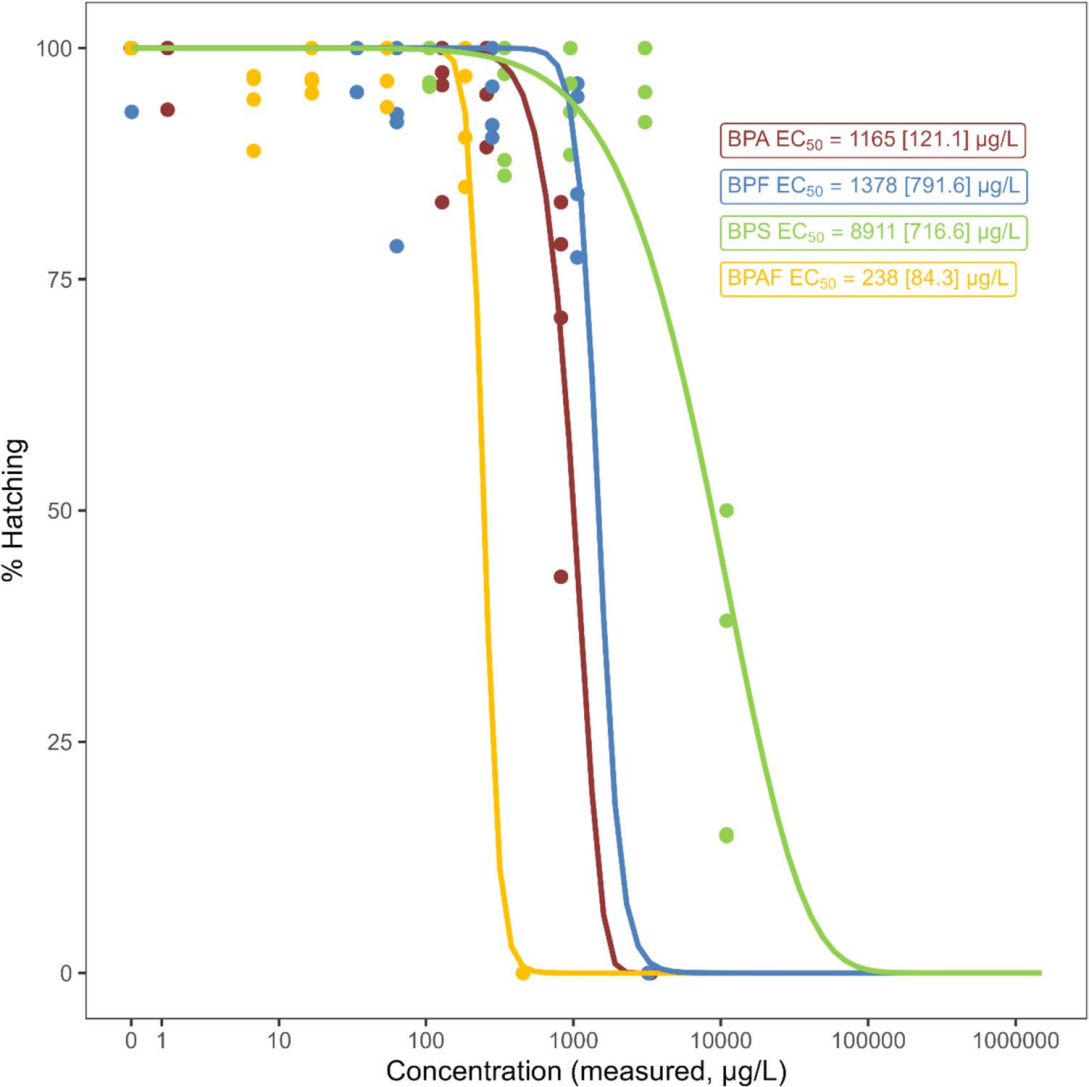
Effect of bisphenols on the hatching of freshwater snail (*Planorbella pilsbryi*) embryos. EC_50_ values (estimate [standard error]) were modelled by non-linear regression using the *drc* statistical package v.1.1.456 (Ritz et al. 2015) in R v .4.1.0 (R Core Team 2021)

For all tests, snail embryo hatching was affected by bisphenols at the highest exposure concentration (3355 µg/L BPA, 3222 µg/L BPF, 10,945 µg/L BPS, or 457 µg/L BPAF; Fig. 3). The EC_50_, EC_25_, and EC_10_ values for hatching are summarized in Table S6. The toxicity ranking was BPAF > BPA > BPF > BPS.

## Discussion

In the present study, we assessed the toxicity of BPA and three commonly-used replacement substances, BPF, BPS, and BPAF, in 96-h tests with adult freshwater snails (*Planorbella pilsbryi*) and in 13-day tests with snail embryos. We then assessed the chronic toxicity of the two most toxic chemicals in our study, BPA and BPAF, in 28-day tests with adult snails, in which the hatching and survival of embryos from the F1 generation was monitored over an additional 21 days. The results of the present study indicate that in acute tests with adult snails, reproductive output was the most sensitive endpoint. Using this endpoint, we calculated a 96-h MATC of 1404 µg/L for BPA (Table 6), which is similar to the 96-h LC_50_ of 2.24 mg/L reported for the gonochoric snail *Marisa cornuarieti*s (Mihaich et al. 2009). Although we observed lower reproductive output at the lowest exposure concentration, 0.072 µg/L BPA, a concentration too close to the method detection limit for consistent quantitation, and at 67 µg/L BPA, it is unclear whether these results were coincidental. In subsequent tests, we implemented a 72-h acclimation period, to ensure a balanced distribution of fertile groups among replicates and ensure that every effort had been made to minimize coincidental discrepancies in fertility independent from exposure (OECD 2016).

**Table 6:** Relative toxicity of bisphenols in two life stages of freshwater snails (*Planorbella pilsbryi*) in aqueous tests. *MATC*, maximum acceptable toxicant concentration; *n.t.*, not tested. EC_50_ values (estimate (standard error)) were modelled by non-linear regression using the best-fitting among 3- or 4-parameter log-logistic and Weibull models. Computations were completed using the *drc* statistical package v.1.1.456 (Ritz et al. 2015) in R v .4.1.0 (R Core Team 2021). Concentrations are reported as micrograms per litre (measured)

Life stage	Test duration	Endpoint	BPA	BPF	BPS	BPAF
Adults (F0)	96 h	MATC^1^	1404	1525	> 8590	136
Embryos (F0)	13 days	EC_50_	1165 (121.1)	1378 (794.6)	8911 (716.6)	238 (84.3)
Adults (F0)	28 days	MATC	> 479	n.t.	n.t.	> 49
Juveniles (F1)	21 days	MATC	13	n.t.	n.t.	> 49

^1^The maximum acceptable toxicant concentration (MATC) is the geometric mean of the no-observed effect concentration (NOEC) and the lowest-observed effect concentration (LOEC)

^2^A non-monotonic decrease in the number of juveniles produced was observed at 0.13 µg/L BPA but was not used in the calculation of the MATC

Of the three replacement substances tested, BPAF was more toxic than BPA, with a MATC of 136 µg/L in the 96-h adult test (Table 5), and an EC_50_ value of 238 µg/L (hatching) in the 13-day embryo test (Table 6; Table S6), compared to those of 1404 and 1165 µg/L for BPA, respectively. In both adult and embryo tests, the toxicity ranking of the compounds tested was BPAF > BPA > BPF > BPS. For BPF, a MATC of 1525 µg/L was calculated in the 96-h adult snail test, and an EC_50_ value (hatching) of 1378 µg/L was calculated for the embryo test. BPS was the least toxic compound, with a MATC > 8590 µg/L (96-h adult test), and an EC_50_ value of 8911 µg/L (embryo hatching). These relative toxicity rankings are in order of decreasing log *K*_OW_ values reported for these chemicals (Table 1) and are similar to those reported for a 96-h exposure of zebrafish (*Danio rerio*) embryos, with calculated LC_50_ values of 1.6 mg/L (BPAF), 12 mg/L (BPA), 32 mg/L (BPF), and 199 mg/L (BPS) (Moreman et al. 2017). Tišler et al. (2016) also assessed the relative toxicity of BPA, BPF, and BPAF using a bioluminescence test (*Vibrio fischeri*), an algae growth inhibition test (*Desmodesmus subspicatus*), acute and chronic *Daphnia magna* tests, and an acute toxicity test with *D. rerio* embryos; they reported toxicity endpoint values in the low milligram per litre range in short-term tests—BPAF was generally more toxic than BPA (Tišler et al. 2016).

Although numerous studies reported on the impact of BPA on the reproduction and embryonic development of vertebrates, fewer studies have focused on potential effects on freshwater gastropods. When we assessed the toxicity of BPA in a 28-day chronic test, survival, behaviour, and reproductive output of *P. pilsbryi* were unaffected at concentrations up to 479 µg/L, but the hatching success of eggs laid at the end of the experiment decreased, with a MATC of 13 µg/L, which suggests potential effects on embryo development and/or reduced viability of the egg masses. However, when we exposed naïve egg masses (F0 generation) to BPA, we calculated a 13-day EC_50_ (hatching) of 1165 µg/L (Table 6). The 100-fold difference in sensitivity between egg masses from F0 (from unexposed parents) and F1 (from exposed parents) generations suggests decreased egg mass viability or potential multi-generation effects. In a 6-month chronic study with *M. cornuarietis*, no effects of BPA were detected on the survival, reproductive output, or the hatching of juveniles at concentrations up to 640 µg/L (Forbes et al. 2008). In contrast, increased embryo production was reported in a 28-day exposure of the parthenogenetic New Zealand mud snail (*Potamopyrgus antipodarum*) to BPA, with a NOEC of 20 µg/L (Sieratowicz et al. 2011). We did not observe any greater effects of BPAF on the F1 generation than on the naïve egg masses. Declines in the reproduction of freshwater snails could significantly impact ecosystem populations, communities, and health.

The role and contribution of vertebrate-like hormones to molluscan reproduction has been the topic of decades of invertebrate research, and remains the subject of considerable debate (Fodor et al. 2022; Fodor and Pirger 2022; Scott 2013, 2012). Although the research presented here did not attempt to resolve this question, in a follow-up study, we extracted the metabolome and lipidome of adult snails chronically exposed to BPA, with the goal to identify pathways affected by the exposure (McNabney et al., in preparation).

To further assess the effects of BPA and its replacement substances on early life stages of freshwater snails, we assessed toxicity in developing snail embryos, and measured EC_50_ values (hatching) varying between 238 µg/L (BPAF) and 8911 µg/L (BPS; Fig. 3). Relative toxicity was similar to that observed in the 96-h tests with adult snails. Other studies into the effects of BPA on gastropod development reported effects within approximately an order of magnitude: Liu et al. (2011) reported a 96-h EC_50_ value of 1 µg/L BPA based on the completion of larvae metamorphosis in the abalone *Haliotis diversicolor supertaxa* (Liu et al. 2011), and exposure to 500 µg/L BPA significantly affected embryo hatchability of the hermaphroditic snail *Physa acuta* (Sánchez-Argüello et al. 2012), while we report a 13-day EC_50_ (hatching) value of 1165 µg/L (Fig. 3; Table 6). To the best of our knowledge, this study is the first to report on the relative toxicity of alternative substances BPF, BPS, and BPAF in freshwater gastropods.

The bioconcentration of bisphenols in freshwater snails was generally lower than the values predicted by EPI Suite (Table 1), except for BPS, which corresponded with predicted values (BCF of 3.0 to 5.4 L/kg [Table 2] vs. 3.5 L/kg predicted by EPI Suite [Table 1]). Similarly, BCFs ranging from 0.1 to 7.6 L/kg were reported for BPS in studies with zebrafish (*Danio rerio*) (Moreman et al. 2017; Yang et al. 2022). We calculated BCFs of 8.3 (96h, Table 2) and 13.9 L/kg (28 days, Table 3) for BPA, while EPI Suite predicted a BCF of 173.8 L/kg (Table 1). Our calculated values are within the lower range of those reported in various studies with fish and bivalves (1.7 to 182 L/kg), and lower than those reported in amphibians and phytoplankton (summarized in Corrales et al. 2015). Surprisingly, the BCF calculated for BPF, 2.5 L/kg (Table 2), was lower than that of BPS, and considerably lower than the predicted value of 28.2 L/kg (Table 1). Moreman et al. (2017) and Yang et al. (2022) reported BCFs of 17.8 to 44.7 L/kg for BPF. The BCFs calculated for BPAF varied between 12.7 (Table 2) and 64.8 (Table 3). Laboratory studies reported BCFs of 5.2 to 102.3 L/kg (Moreman et al. 2017; Shi et al. 2016; Yang et al. 2022). In general, the calculated BCFs for the substances of interest appear to be within the lower range of values reported for aquatic organisms. As *P. pilsbryi* is a pulmonate snail, lower bioconcentration could be due to decreased waterborne exposure compared to organisms breathing through gills, a hypothesis that would need to be investigated further. In summary, the BCFs for BPA, BPF, and BPAF were lower than predicted by EPI Suite yet within the lower range of values reported by other studies, while the BCF for BPS was in alignment with the predicted value, and thus considerably greater than expected compared to the other compounds with greater log *K*_OW_.

Based on the results of the present study, concentrations of bisphenols reported in surface water are unlikely to cause adverse effects to freshwater snails, yet predators could incur additional exposure through consumption of contaminated prey. Wang et al. (2017) assessed the bioaccumulation and trophic magnification of several bisphenols in aquatic systems and reported a positive association between their log *K*_OW_ and their bioaccumulation factor (Wang et al. 2018). Bisphenol A, F, and S did not appear to biomagnify significantly, but other compounds, including BPAF, BPC, and BPZ, did (Gu et al. 2016). We calculated BCFs of 8.3 and 13.9 L/kg for BPA, lower than the value predicted by modelling (Table 1). The BCF of BPF was 2.5 L/kg; that of BPS, 3.0 to 5.4 L/kg; and that of BPAF, 12.7 to 64.8 L/kg. Similarly, Moreman et al. (2017) reported BCFs of 3.1 and 4.5 L/kg for BPA, 17.8 L/kg for BPF, 0.067 L/kg for BPS, and 5.3 L/kg for BPAF in zebrafish embryos after a 96-h exposure; their values appear within the general range of our data.

In summary, the results of the present study confirm that BPA was slightly more toxic than alternatives BPF and BPS, but that BPAF was more toxic than BPA, in 96-h acute tests with adult file ramshorn snails (*Planorbella pilsbryi*) and in 13-day tests with developing snail embryos. The reported relative toxicity was consistent with the hydrophobicity (log *K*_OW_) of each compound. Chronic testing with BPA revealed that the eggs from the F1 generation were less viable or considerably more sensitive, with effects on hatching and survival at concentrations of 42 µg/L (MATC) even though we did not observe significant effects on the survival, growth, and reproductive output of the adults at a concentration up to 629 µg/L BPA. Sensitivity could not be explained by the earlier life stage, as snail embryos produced from naïve snails had EC_50_ values similar to those of the adult snails. Though the effects observed were generally at concentrations beyond those reported in surface water, the presence of an overlap between exposure and effects concentrations and the potential for mixtures of bisphenols to exert additive effects warrant further research.

## Supplementary Information

Below is the link to the electronic supplementary material.

Supplementary file1 (PDF 295 KB)

## Data Availability

Data supporting the findings of this research are available on the Government of Canada’s Open Government: Toxicity and bioconcentration of Bisphenol A alternatives on the freshwater gastropod Planorbella pilsbryi: https://open.canada.ca/data/en/dataset/68adbe49-d3d5-4a24-827f-4bdf9f51156d.
