# Supplementary material for: Toxicity and bioconcentration of bisphenol A alternatives in the freshwater pulmonate snail Planorbella pilsbryi
Source: Environ Sci Pollut Res Int. 2025 Feb 5;32(9):5186–99. doi: 10.1007/s11356-025-36019-w (PMC11868244; doi:10.1007/s11356-025-36019-w)
Supplement: Supplementary file 1 — Supplementary file1 (PDF 295 KB) [file 11356_2025_36019_MOESM1_ESM.pdf]

Supplemental Material

Toxicity and bioconcentration of Bisphenol A alternatives in the freshwater pulmonate snail *Planorbella pilsbryi*

Ève AM Gilroy[1*], Karyn B Robichaud[1], Maria Villella[1], Kara Chan[1], David WG McNabney[1], Carmen Venier[2], Victor Pham-Ho[1], Émilie C Montreuil Strub[1], Shelby A Ravary[1], Ryan S Prosser[2] and Stacey A Robinson[3]

[1] Aquatic Contaminants Research Division, Environment and Climate Change Canada, Burlington, ON
[2] School of Environmental Sciences, University of Guelph, Guelph, ON
[3] Ecotoxicology and Wildlife Health Division, Environment and Climate Change Canada, Ottawa, ON

[*]Corresponding author: eve.gilroy@ec.gc.ca

Table S1 Water quality parameters (mean ± standard deviation (*n*)) collected throughout the toxicity studies with adult freshwater snails *Planorbella pilsbryi*.

Test Duration	Compound	pH	Temperature (°C)	Dissolved Oxygen (mg/L)	Conductivity (µS/cm)	Ammonia (mg/L)
96 h	BPA	7.8 ± 0.44 (19)	23.0 ± 0.3 (19)	5.5 ± 3.15 (19)	401 ± 36 (19)	1.3 ± 2.24 (18)
	BPF	7.8 ± 0.23 (20)	21.4 ± 0.2 (20)	6.8 ± 1.92 (20)	358 ± 34 (20)	0.2 ± 0.90 (20)
	BPS	7.9 ± 0.17 (20)	21.9 ± 0.5 (20)	7.6 ± 1.01 (20)	369 ± 30 (20)	0.5 ± 2.06 (19)
	BPAF	7.6 ± 0.23 (25)	23.1 ± 0.15 (25)	6.6 ± 2.33 (25)	346 ± 64 (20)	0.2 ± 0.41 (25)
28 d	BPA	7.8 ± 0.18 (72)	23.0 ± 0.43 (72)	6.8 ± 1.55 (72)	388 ± 23 (72)	0.4 ± 1.28 (72)
	BPAF	8.0 ± 0.20 (64)	22.4 ± 0.43 (64)	8.1 ± 0.72 (64)	374 ± 20.7 (64)	1.0 ± 1.80 (64)

Table S2. Ion transitions and internal standards used for each analyte for chemical analysis of aqueous samples of Bisphenols A (BPA), F (BPF), S (BPS) and AF (BPAF) completed by the National Laboratories for Environmental Testing (Burlington, ON).
Note: quantitation/qualifier ion ratios were used to determine if interferences are present in the quantitation ion signal. Analyses were completed in negative electrospray mode using an isocratic gradient (70% Acetonitrile: 30% Water). The full method duration was 3.5 minutes.

**Instrument 1 (2019-2021)**	Waters Xevo TQ-S UHPLC-MS/MS
**Source temperature (°C)**	150
**Desolvation temperature (°C)**	400
**Desolvation gas flow (L/hr)**	800
**Cone gas flow (L/hr)**	150
**Collision gas flow (mL/min)**	0.07
**Capillary voltage (kV)**	3

**Target Analyte**	**Quantitation & Qualifier Ion Pairs (m/z)**	**Internal Standard**	**Ratio Quantitation/Qualifier peak area (± limit*)**	**V_f (v)**	**Retention Time (min)**
BPA	227.1 – 212.0	$^{13}C_{12}$ BPA	2.9	18	0.85
	227.1 – 133.0			26	
$^{13}C_{12}$ BPA	239.0 – 224.0	-	Internal standard	18	
BPF	199.1 – 105.1	$^{13}C_{12}$ BPF	-	24	0.78
$^{13}C_{12}$ BPF	211.0 – 99.1	-	Internal standard	22	
BPS	249.0 – 108.0	$^{13}C_{12}$ BPS	3.0	28	0.70
	219.0 – 156.0			24	
$^{13}C_{12}$ BPS	261.1 – 114.0	-	Internal standard	26	
BPAF	335.1- 265.1	$^{13}C_{12}$ BPAF	-	24	0.95
$^{13}C_{12}$ BPAF	347.2- 277.1	-	Internal Standard	22	

**Instrument 2 (2021-present)**	Sciex 6500+ QTRAP UHPLC
**Curtain gas flow (PSI)**	20.0
**Ion Source temperature (°C)**	450
**Ion source gas 1 (PSI)**	50.0
**Ion source gas 2 (PSI)**	50.0
**Ion spray voltage (V)**	-4,000
**Entrance potential (V)**	-10
**Collision cell exit potential**	-11

**Target Analyte**	**Quantitation* & Qualifier Ion** Pairs (m/z)**	**Internal Standard**	**Ratio Quantitation/Qualifier peak area (± limit*)**	**Retention Time (min)**	**Collision Energy (V)**	**Declustering Potential (V)**
BPA	227.0 – 212.0*	$^{13}C_{12}$ BPA	0.22	0.92	-26	-100
	227.0 – 133.0**					
$^{13}C_{12}$ BPA	239.0 – 224.0	-	Internal standard			
PBF	199.0 – 93.0*	$^{13}C_{12}$ BPF	4.09	0.84	-30	-97
	199.0- 105.0**					
$^{13}C_{12}$ BPF	211.0- 99.0	-	Internal standard			
BPS	249.0 – 108.0*	$^{13}C_{12}$ BPS	0.23	0.77	-33	-147
	249.0- 156.0**					
$^{13}C_{12}$ BPS	261.0 – 114.0	-	Internal standard			
BPAF	335.0- 265.0*	$^{13}C_{12}$ BPAF	0.00	1.03	-29	-133
	335.0- 197.0**					
$^{13}C_{12}$ BPAF	347.0- 277.0	-	Internal standard			

Table S3. Analytes, ions and quantification references for chemical analysis of tissue samples of Bisphenols A (BPA), B (BPB), E (BPE), F (BPF), S (BPS) and AF (BPAF), completed by SGS Axys (Sydney, BC), using Method MLA-113. The full method duration was 15 minutes.

Target Analyte	Quantitation* & Qualifier Ion** Pairs (m/z)	Typical Retention Time (Minutes)	Internal Standard
**Analytes**			
BPA	227.211 – 133.041*<br>227.211 – 212.051**	5.92	$^{13}C_{12}$-BPA
BPB	241.29 – 212.028*<br>241.29 – 226.207**	6.29	$^{13}C_{12}$-BPB
BPE	213.259 – 197.967*<br>213.259 – 118.971**	5.72	$^{13}C_{12}$-BPF
BPF	199.18 – 92.942*<br>199.18 – 104.984**	5.54	$^{13}C_{12}$-BPF
BPS	249.19 – 107.917*<br>249.19 – 91.963**	4.71	$^{13}C_{12}$-BPS
BPAF	335.09 –265.09*<br>335.09 – 196.988**	5.95<br>5.89	$^{13}C_{12}$-BPAF
**Surrogate Standards**			
$^{13}C_{12}$-BPF	211.092	5.54	D_6-BPA
$^{13}C_{12}$-BPA	239.187	5.92	D_6-BPA
$^{13}C_{12}$-BPAF	347.258	5.95	D_6-BPA
$^{13}C_{12}$-BPB	253.203	6.3	D_6-BPA
**Recovery standard**			
D_6-Bisphenol A	233.312	5.90	External

* Quantitation ions were used for quantification.

**Qualifier ions represent confirmation product ions and may be used in instances of interference. For compounds having two MRMs, ion ratios were monitored and compared with the ion ratio generated from the opening calibration run. An interim ion ratio criterion of 50% was used for concentrations that produce S:N ratios greater than 10 in both MRMs.

Table S4. Survival and frequency of activity (mean [standard deviation]) of the freshwater snail *Planorbella pilsbryi* exposed to Bisphenol A, F, S and AF during aqueous 96-h static tests. Asterisks denote significant differences from pooled controls.

Chemical Tested	Treatment/Nominal Concentration µg/L)	Treatment/Measured Concentration (µg/L)	Survival (%)	Activity (%)
Bisphenol A	Control	Control	90 [11.0]	90 [3.3]
	Solvent Control	Solvent Control	93 [10.3]	95 [5.5]
	0.01	0.072	87 [16.3]	89 [6.9]
	0.1	0.086	80 [17.9]	87 [13.5]
	1	0.65	87 [16.3]	91 [6.0]
	10	3.8	87 [24.2]	86 [8.3]
	100	67	83 [19.7]	89 [5.9]
	1000	692	97 [8.2]	89 [9.4]
	5000	2843	30 [21.0]*	3 [8.2]*
Bisphenol F	Control	Control	100 [0]	97 [8.2]
	Solvent Control	Solvent Control	100 [0]	99 [2.0]
	0.01	0.012	93 [10.3]	88 [8.2]
	0.1	0.16	93 [16.3]	93 [8.2]
	1	0.41	83 [23.4]	81 [24.2]
	10	4.4	97 [8.2]	96 [8.0]
	100	46	100 [0]	97 [4.1]
	1000	476	100 [0]	93 [6.9]
	10,000	1930	97 [8.2]	70 [12.2]
	BPA (10,000)	BPA (9285)	0 [0]*	0 [0]*
Bisphenol S	Control	Control	100 [0]	100 [0]
	Solvent Control	Solvent Control	100 [0]	99 [1.6]
	0.01	0.12	100 [0]	100 [0]
	0.1	0.37	100 [0]	99 [1.6]
	1	0.88	100 [0]	99 [2.1]
	10	8.3	100 [0]	98 [2.2]
	100	89	100 [0]	99 [2.1]
	1000	843	100 [0]	99 [1.6]
	10,000	8590	100 [0]	97 [4.1]
	BPA (10,000)	BPA (7035)	33 [51.6]*	0 [0]*
Bisphenol AF	Control	Control	100 [0]	100 [0]
	Solvent Control	Solvent Control	100 [0]	100 [0]
	0.01	0.48	100 [0]	100 [0]
	0.1	0.23	100 [0]	100 [0]
	1	2	100 [0]	100 [0]
	10	5.2	100 [0]	98 [4.1]
	100	37	100 [0]	100 [0]
	1000	486	100 [0]	58 [26.4]
	3200	1930	0 [0]*	0 [0]*
	BPA (10,000)	BPA (7190)	0 [0]*	2 [4.1]*

Table S5. Survival, frequency of activity, reproductive output, length, weight, number of juveniles, and day to first hatch of F1 juveniles (mean [standard deviation]) of freshwater snails *Planorbella pilsbryi* exposed to Bisphenol A (BPA) and Bisphenol AF (BPAF) during aqueous 28 (adult) + 21 (F1 embryos) d static renewal tests. Values followed by an asterisk denote significant difference from pooled controls. § denotes a significant difference from the control, but not the solvent control or pooled controls.

**Chemical Tested**	**Treatment/Measured Concentration (µg/L)**	**Survival (%)**	**Activity (%)**	**Reproductive Output (egg masses/snail/week)**	**Length (mm)**	**Weight (g)**	**Number of juveniles**	**Day to first hatch (d post-exposure)**
BPA	Control	90 [11.0]	95 [5.4]	4.5 [0.95]	16.3 [0.29]	1.18 [0.054]	362 [106.1]	8.0 [0.00]
	Solvent Control	93 [10.3]	93 [3.3]	4.7 [1.26]	16.3 [0.23]	1.18 [0.047]	234 [135.0]	8.2 [0.41]
	0.014	100 [0]	92 [2.9]	4.7 [0.40]	16.0 [0.30]	1.15 [0.085]	195 [48.1]	9.0 [0.63]
	0.13	100 [0]	94 [4.1]	4.1 [1.00]	15.8 [0.66]	1.11 [0.088]	107 [114.5]§	12.7 [5.24]
	0.45	97 [8.2]	94 [2.7]	4.8 [0.79]	16.2 [0.29]	1.17 [0.058]	241 [176.2]	11.7 [5.28]
	3.7	93 [10.3]	91 [5.9]	4.3 [0.30]	16.4 [0.55]	1.20 [0.115]	202 [166.6]	9.7 [3.2]
	42	97 [8.2]	94 [5.8]	4.4 [1.09]	16.4 [0.30]	1.20 [0.052]	78 [85.1]*	11.2 [4.26]
	479	100 [0]	92 [3.1]	4.1 [1.01]	16.1 [0.33]	1.13 [0.064]	36 [54.9]*	16.0 [5.73]*
BPAF	Control	93 [10.3]	100 [0]	8.1 [1.48]	16.5 [0.75]	0.98 [0.127]	263 [175.0]	9.2 [1.17]
	Solvent Control	97 [8.2]	100 [0]	8.0 [0.82]	16.7 [0.86]	1.01 [0.143]	189 [151.9]	7.7 [0.82]
	0.27	93 [10.3]	99 [2.3]	8.3 [0.75]	16.6 [0.88]	1.00 [0.166]	176 [181.9]	9.5 [2.59]
	0.07	97 [8.2]	100 [0]	8.2 [1.13]	16.4 [0.84]	0.98 [0.155]	163 [101.4]	8.3 [1.37]
	0.56	93 [10.3]	99 [1.0]	8.6 [1.47]	16.4 [0.91]	0.95 [0.141]	182 [147.5]	8.2 [0.98]
	5	100 [0]	100 [0]	8.0 [0.91]	16.4 [0.97]	0.98 [0.156]	152 [112.8]	9.8 [2.93]
	49	93 [10.3]	99 [1.0]	8.4 [0.71]	16.7 [0.89]	0.97 [0.160]	221 [165.8]	9.2 [2.93]
	547	97 [8.2]	100 [0]	8.0 [1.10]	16.8 [1.16]	1.05 [0.178]	98 [104.9]	9.8 [4.02]

Table S6. Toxicity of Bisphenols A, F, S and AF to *Planorbella pilsbryi* embryos in aqueous 13-d static renewal tests (embryos), expressed as ECx values for hatching. Concentrations are reported as µg/L (**measured**). All calculations were completed using the *drc* statistical package v.1.1.456 (Ritz et al., 2015) in R v .4.1.0 (R Core Team, 2021). The best-fitting among 3- or 4-parameter log-logistic and Weibull models was selected.

Compound	Effect Measure	Estimate [SE]	95% Confidence Interval
Bisphenol A	EC_{10}	402 [76.2]	244.3-560.4
	EC_{25}	709 [66.4]	571.4-846.8
	EC_{50}	1165 [121.1]	913.4-1415.7
Bisphenol F	EC_{10}	1038 [73.5]	886.3-1,191.3
	EC_{25}	1196 [313.1]	547.0-1845.8
	EC_{50}	1378 [794.6]	-263.9-3019.6
Bisphenol S	EC_{10}	4466 [1,399.3]	1564.1-7368.3
	EC_{25}	6455 [1,200.5]	3965.3-8944.6
	EC_{50}	8911 [716.6]	7424.9-10,397.4
Bisphenol AF	EC_{10}	192 [12.1]	167.2-217.4
	EC_{25}	214 [44.0]	122.5-305.2
	EC_{50}	238 [84.3]	63.0-412.6

References

R Core Team, 2021. R: A Language and Environment for Statistical Computing. Vienna, Austria. https://www.R-project.org/

Ritz, C., Baty, F., Streibig, J.C., Gerhard, D. 2015. Dose-Response Analysis using R. PLoS ONE 10:e0146021. https://doi.org/10.1371/journal.pone.0146021
